# Supplementary material for: Insights into Drug Cardiotoxicity from Biological and Chemical Data: The First Public Classifiers for FDA Drug-Induced Cardiotoxicity Rank
Source: J Chem Inf Model. 2024 Feb 1;64(4):1172–86. doi: 10.1021/acs.jcim.3c01834 (PMC10900289; doi:10.1021/acs.jcim.3c01834)
Supplement: Supplementary file 1 — ci3c01834_si_002.pdf [file ci3c01834_si_002.pdf]

Supporting Information

Insights into Drug Cardiotoxicity from Biological and Chemical Data: The First Public Classifiers for FDA DICTrank

Srijit Seal[1], Ola Spjuth[2], Layla Hosseini-Gerami[3], Miguel García-Ortegón[4], Shantanu Singh[1], Andreas Bender[4], Anne E. Carpenter[1]*

[1]Imaging Platform, Broad Institute of MIT and Harvard, Cambridge, MA 02142, US

[2]Department of Pharmaceutical Biosciences and Science for Life Laboratory, Uppsala University, Box 591, SE-75124, Uppsala, Sweden

[3] Ignota Labs, County Hall, Westminster Bridge Rd, SE1 7PB, London, United Kingdom

[4] Yusuf Hamied Department of Chemistry, University of Cambridge, Lensfield Rd, CB2 1EW, Cambridge, United Kingdom

Documentation: https://broad.io/DICTrank_Predictor

Code: https://github.com/srijitseal/DICTrank

Datasets: 10.6084/m9.figshare.24312274

*Correspondence:

seal@broadinstitute.org

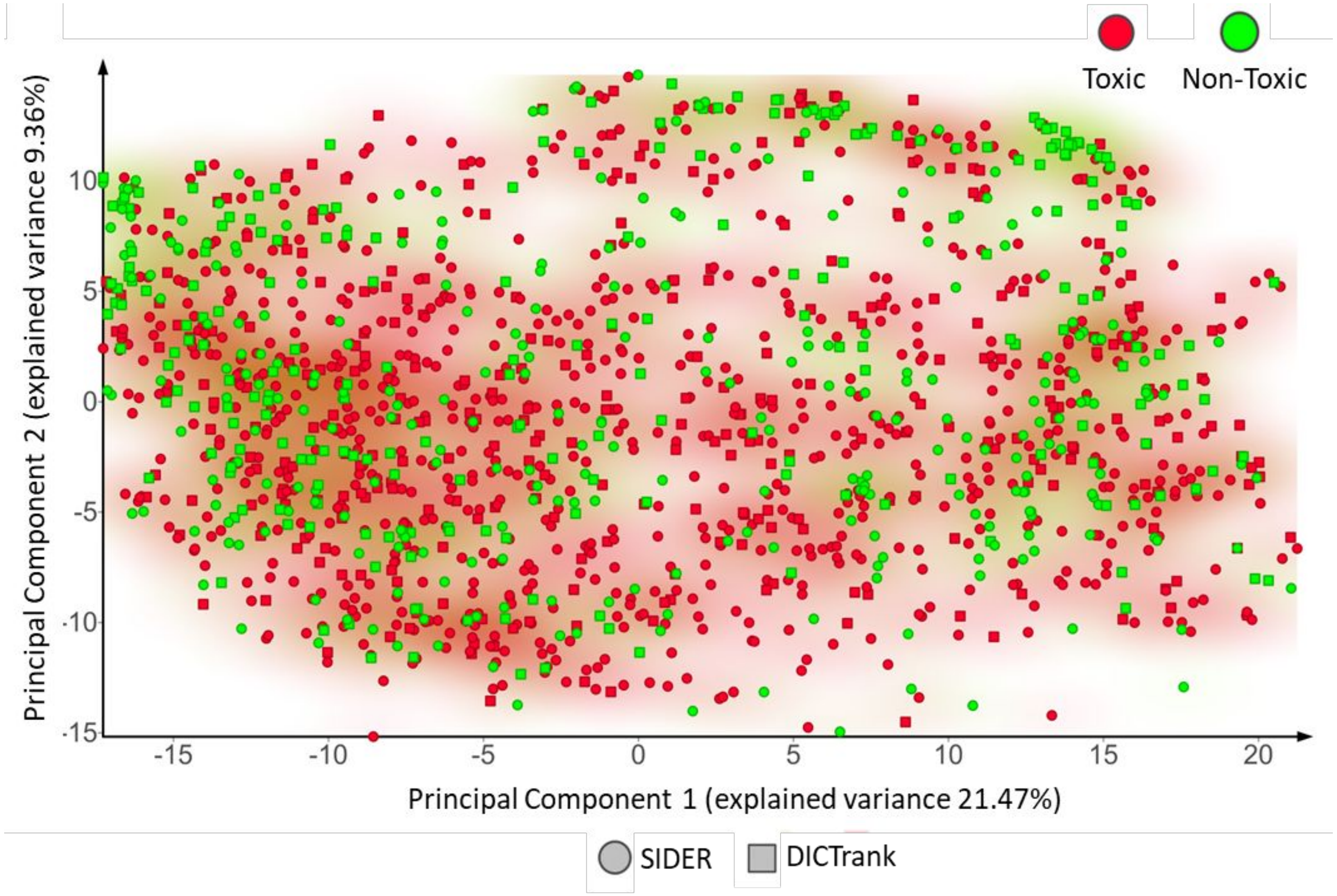

Toxic
Non-Toxic
Principal Component 2 (explained variance 9.36%)
Principal Component 1 (explained variance 21.47%)
10
5
0
-5
-10
-15
-15
-10
-5
0
5
10
15
20
SIDER
DICTrank

Figure S1: The chemical space of all 1020 DICTrank compounds and the remaining 546 unique compounds in SIDER as defined by FragFP fingerprints (chemical fingerprints) in a principal component analysis. SIDER and DICTrank compounds fairly overlap (with the PCA explained variance of 21.47%).

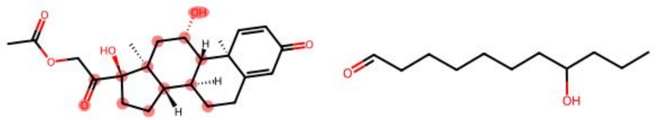

prednisolone acetate

['Cardiac disorders']

Additional Data: ['approved']

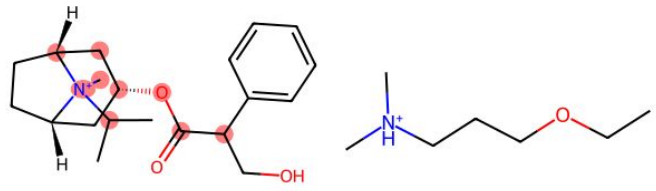

ipratropium

['Cardiac disorders']

Additional Data: ['approved', 'experimental']

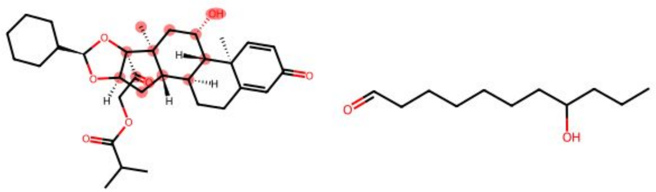

ciclesonide

['Cardiac disorders']

Additional Data: ['approved', 'investigational']

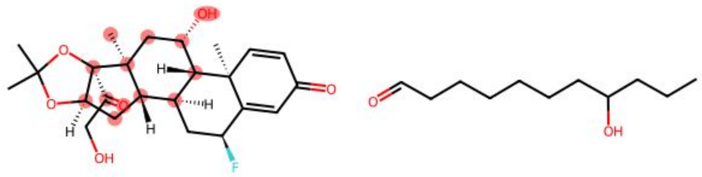

flunisolide

['Cardiac disorders']

Additional Data: ['approved', 'investigational']

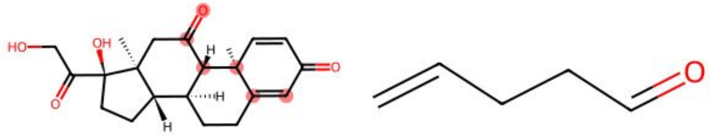

prednisone

['Cardiac disorders']
Additional Data: ['approved']

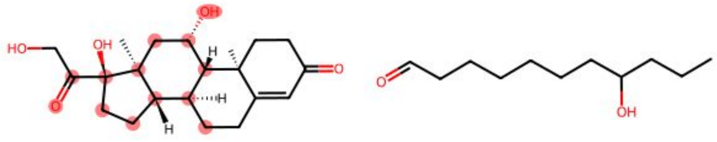

hydrocortisone

['Cardiac disorders']
Additional Data: ['approved']

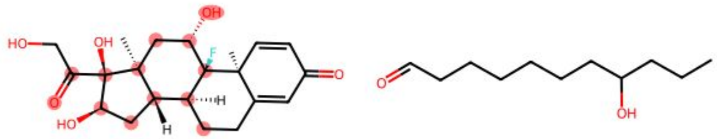

triamcinolone

['Cardiac disorders']
Additional Data: ['approved']

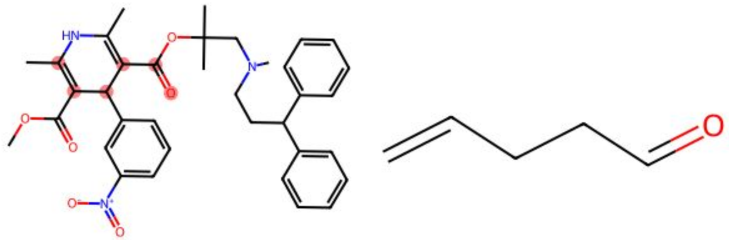

lercanidipine

['Cardiac disorders']
Additional Data: ['approved', 'investigational']

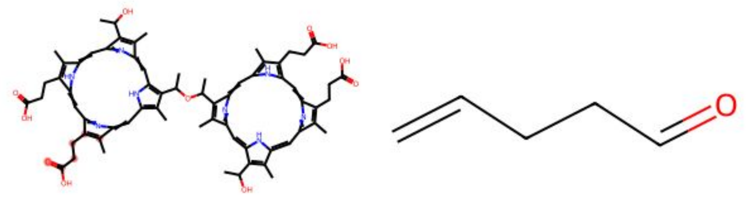

dihematoporphyrin ether

['Cardiac disorders']
Additional Data: ['investigational']

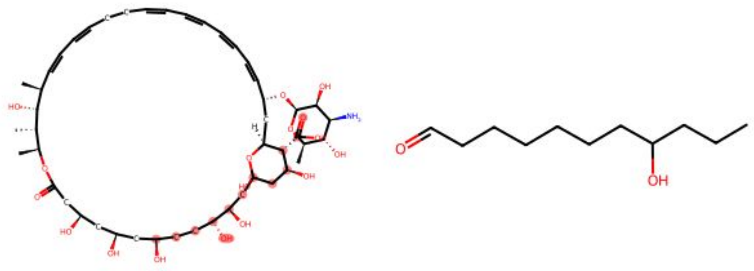

nystatin

['Cardiac disorders']
Additional Data: ['approved']

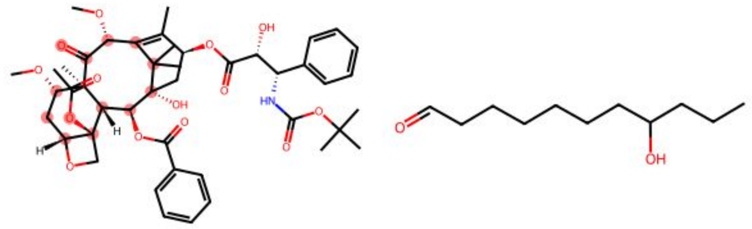

cabazitaxel

['Cardiac disorders']
Additional Data: ['approved']

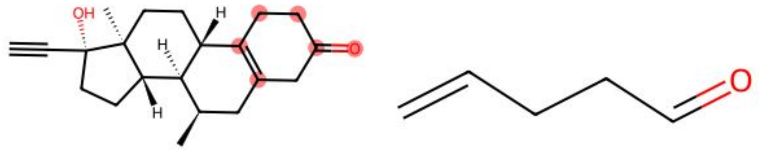

tibolone

['Cardiac disorders']
Additional Data: ['approved', 'investigational']

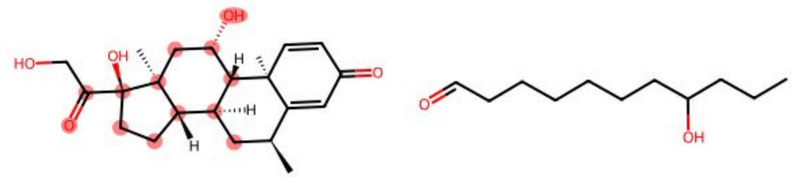

methylprednisolone

['Cardiac disorders']

Additional Data: ['approved']

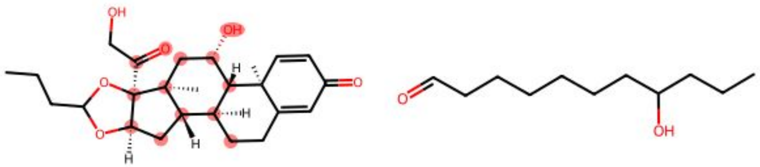

budesonide

['Cardiac disorders']

Additional Data: ['approved']

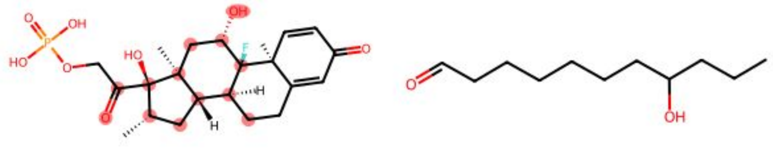

betamethasone phosphate

['Cardiac disorders']

Additional Data: ['approved']

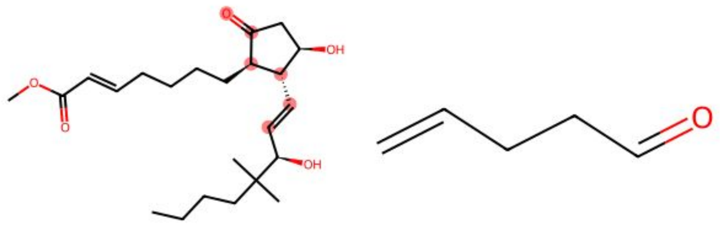

gemeprost

['Cardiac disorders']

Additional Data: ['approved', 'withdrawn']

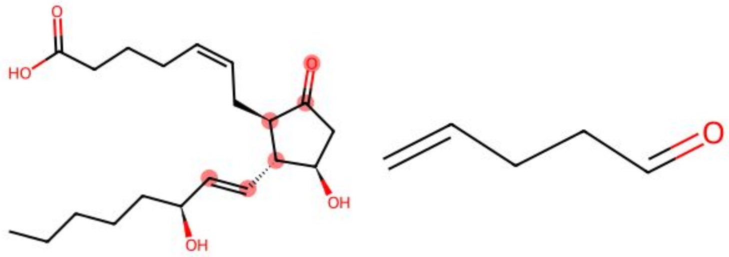

dinoprostone

['Cardiac disorders']
Additional Data: ['approved']

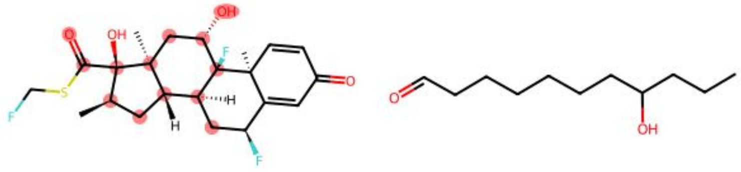

fluticasone

['Cardiac disorders']
Additional Data: ['approved', 'experimental']

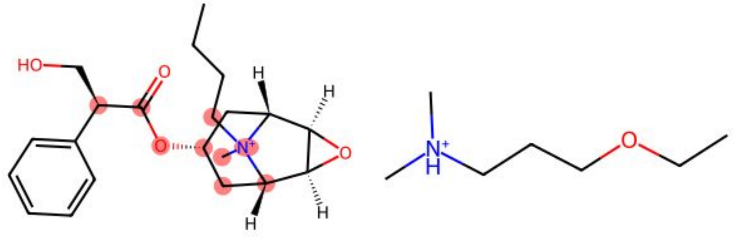

butylscopolamine

['Cardiac disorders']
Additional Data: ['approved', 'investigational']

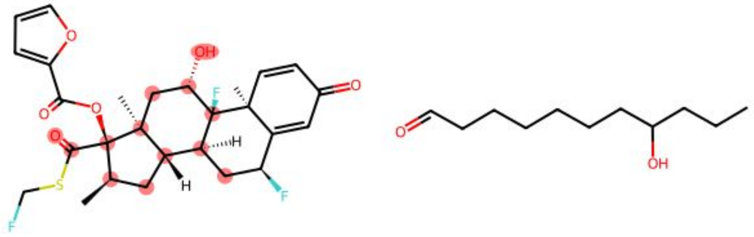

fluticasone furoate

['Cardiac disorders']
Additional Data: ['approved']

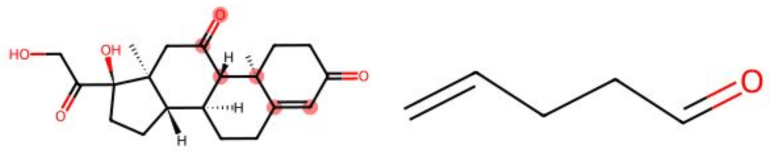

cortisone

['Cardiac disorders']
Additional Data: ['experimental']

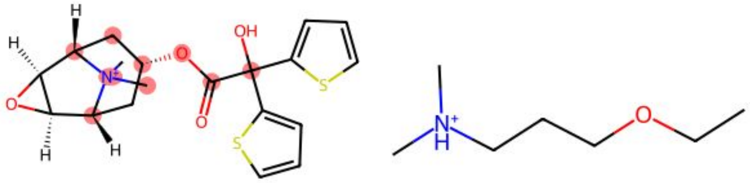

tiotropium

['Cardiac disorders']
Additional Data: ['approved']

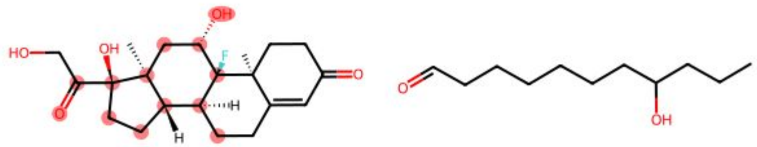

fludrocortisone

['Cardiac disorders']
Additional Data: ['approved', 'investigational']

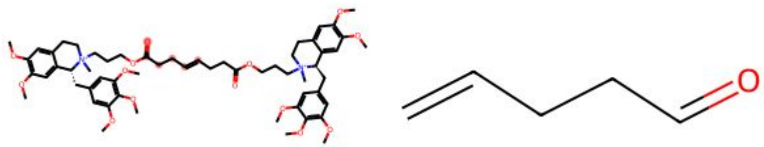

mivacurium

['Cardiac disorders']
Additional Data: ['approved']

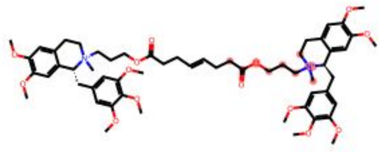

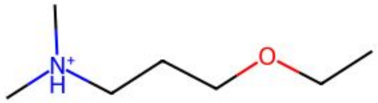

mivacurium

['Cardiac disorders']

Additional Data: ['approved']

Figure S2: Hits from DrugBank compounds containing structural alerts from the top 6 presented in Figure 4(b).

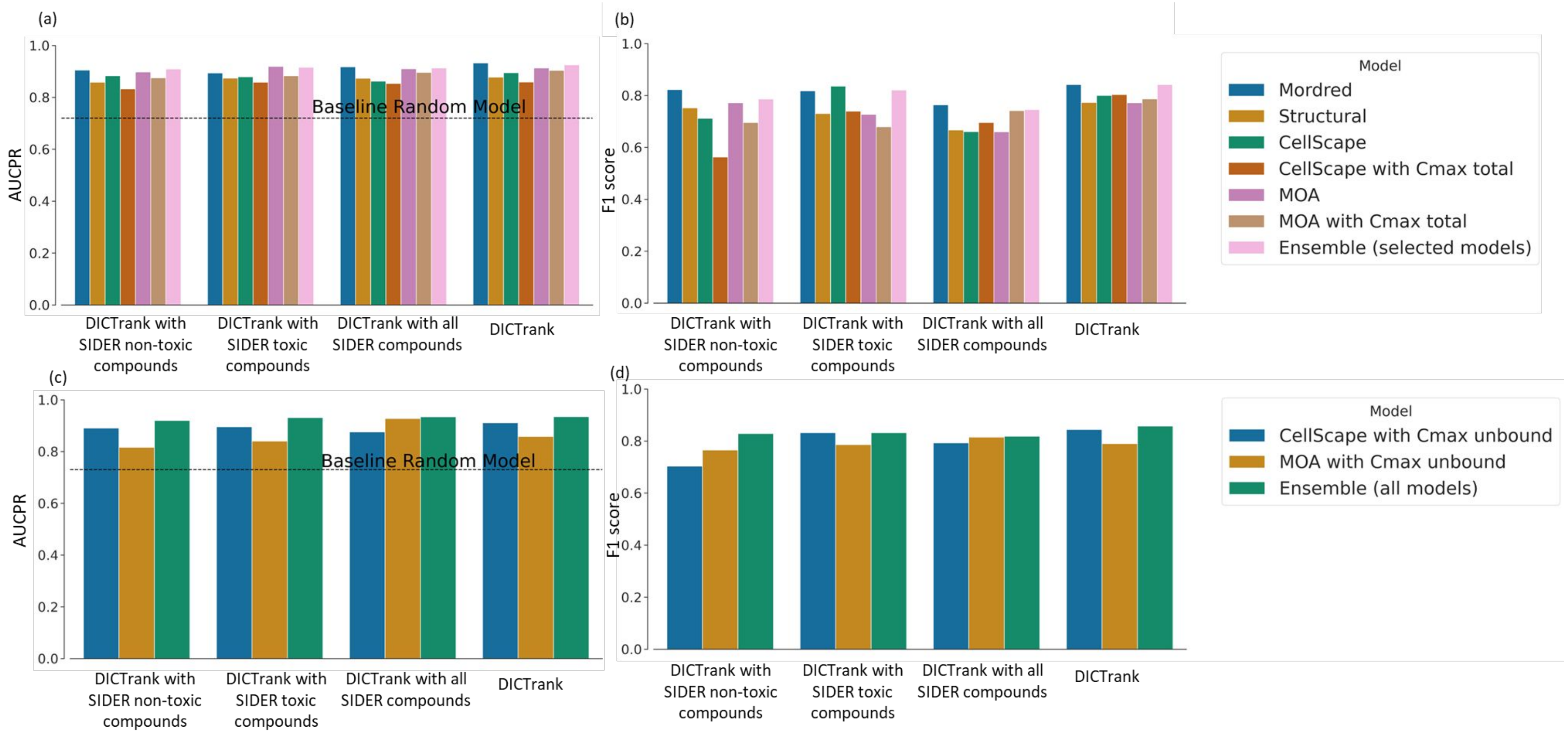

Figure S3: Evaluation metrics for models developed in this study using a larger external test set of 90 compounds evaluated using (a) AUCPR and (b) F1 scores, and for models using a subset of the external test set with 78 compounds evaluated using (c) AUCPR and (d) F1 scores
